# Research on correlation between dynamic resilient modulus and *CBR* of coarse-grained chlorine saline soil

**DOI:** 10.1038/s41598-024-54538-3

**Published:** 2024-02-28

**Authors:** Miaoyi Deng, Jinshan Wang, Xiangyang Wang, Xiangbing Xie, Kaiwei Wang, Yahui He

**Affiliations:** 1 School of Civil and Environmental Engineering, Zhengzhou University of Aeronautics, Zhengzhou, 450046 China; 2 School of Civil Engineering, Qingdao University of Technology, Qingdao, 266520 China; 3 Road and Bridge Institute, Guangdong Jiaoke Testing Co., Ltd., Guangzhou, 510550 China

**Keywords:** California bearing ratio (*CBR*), Coarse-grained chlorine saline soil, Dynamic resilient modulus, Evolution law, Prediction model, Engineering, Materials science

## Abstract

A large area of coarse-grained saline soil is distributed in saline soil areas, and chlorine saline soil with a high salt content is a typical representative. The dynamic resilient modulus was accurately predicted using the California-bearing ratio (*CBR*) value to determine the relationship between the dynamic resilient modulus of coarse-grained chloride saline soil and its *CBR* value. Indoor dynamic triaxial tests and *CBR* tests were conducted to investigate the evolution of the dynamic resilient modulus (*M*_R_) and *CBR* of coarse-grained chlorine saline soil under the influence of the stress level, water content, and salt content. The test results showed that the dynamic resilient modulus increased with an increase in the confining pressure and bulk stress and decreased as the deviator stress increased; however, the *CBR* increased with an increase in the corresponding unit pressure. The higher the salt and water contents, the more obvious the influence of stress on the dynamic resilient modulus and *CBR* value. Under the same stress level, the decrease in the dynamic resilient modulus and *CBR* gradually increased with increasing salt and moisture content, and the effect of salt tended to be more significant than that of water. Based on the correlation between the dynamic resilient modulus and *CBR* revealed by the experiment, a more widely applicable model was selected from the existing theoretical models related to *CBR* for the regression analysis of the test data, and a prediction model of the dynamic resilient modulus based on the *CBR* value was proposed (*M*_R_ = 21.06*CBR*^0.52^). This prediction model had a high correlation coefficient (*R*^2^ = 0.893) and could effectively predict the dynamic resilient modulus of coarse-grained chlorine saline soil using *CBR* values. The results provide a simple and reliable method for determining the design parameters of a coarse-grained saline soil subgrade.

## Introduction

The resilient modulus is not only a mechanical index reflecting the deformation resistance of the roadbed but is also a key parameter in the design of road structure^1–3^. The resilient modulus is the ratio of stress to the corresponding strain of road materials under load and is an effective index for characterizing the elastic deformation characteristics of subgrade soil, which has been widely used in road design^4–6^. Based on an in-depth analysis of the subgrade resilient modulus (*E*_0_), many scholars and research institutions worldwide have found that the dynamic resilient modulus (*M*_R_) is more consistent with the actual mechanical characteristics of roads^7–12^. Simultaneously, the current Chinese subgrade design code clearly indicates that the subgrade filler should take the dynamic resilient modulus of the top surface of the road bed as the design index on the basis of meeting the *CBR* and provides the test method and evaluation standard of the dynamic resilient modulus of subgrade soil^13^. However, owing to the expensive equipment, complex operation, and high cost of dynamic resilient modulus testing, there are some limitations in specific engineering applications. Therefore, if a simple, reliable, and popular *CBR* test method can be used to establish a prediction model for the dynamic resilient modulus based on the *CBR* value^14^, the *CBR* value can be used to accurately predict the dynamic resilient modulus of the subgrade soil, which has important engineering application value.

In view of the correlation between the resilient modulus of subgrade soil and the *CBR* value, researchers at home and abroad have obtained relevant research results. Heukelom and Klomp^15^ obtained a linear relation of *CBR*, *E* = α*CBR,* through a regression analysis based on the field resilient modulus test results of the Shell Company and several other public data. The regression coefficient *α* varied between 5 and 20, generally taking 10 and a safety factor of 2. This relationship has been adopted in several fine-grained soil subgrade design methods^16–19^. Zhao et al.^20^ conducted a large number of field tests on a test road section of silt-medium liquid-limited clay in the Xinyang area, Henan Province, China, and proposed that the relationship between the resilient modulus of the soil foundation and the bearing ratio in this area was *E*_0_ = 3.192*CBR*^0.555^, and the correlation coefficient *R* = 0.9404, indicating a very good correlation between the two. Xiao et al.^21^ took silty clay and silty soil in Jilin Province as research objects, and based on the data of indoor resilient modulus (*E*_0_) test and *CBR* test, obtained the values of resilient modulus of silty clay *E*_0_ ≈ 2.4*CBR* and that of silty soil *E*_0_ ≈ 4.5*CBR* through statistical analysis. These differences were caused by the different properties of silty clay and silty soil. Zhang^22^ used improved silty clay from oil shale waste residue of Jilin Province as the subgrade filler and proposed a dynamic resilient modulus prediction model based on *CBR* value through the *CBR* test and dynamic triaxial test. The model had a high correlation coefficient and strong applicability. Cheng et al.^23^ selected two types of subgrade soil in a seasonally frozen region, such as clay soil and silty soil, to conduct an indoor resilient modulus (*E*_0_) test and *CBR* test and obtained the specific relationship between the resilient modulus of subgrade soil in the seasonal freezing region and *CBR* through linear regression (i.e., low liquid limit clay *E*_0_ = 6.73*CBR*^0.73^, correlation coefficient *R*^2^ = 0.829; low liquid limit silt *E*_0_ = 7.40*CBR*^0.70^, and correlation coefficient *R*^2^ = 0.899). Wu et al.^24^ selected four expressways in Shanxi Province to conduct field tests on the resilient modulus *E*_0_ and the *CBR*. By collating and analyzing the test data, the relationship between the resilient modulus of loess in northern Shanxi and *CBR* was in line with a power exponential relationship, and the expression was *E*_0_ = 5.04*CBR*^0.6–5.15^. The relationship between the loess resilient modulus and *CBR* in northern Shanxi is exponential, and its expression is *E*_0_ = 9.737*e*^0.032CBR^. Cheng^25^ selected the subgrade of typical sections of the Shanping, Guangyuan, and Heyun expressways under construction in the Loess region to conduct in situ resilient modulus and *CBR* tests. Through a linear regression analysis of the measured data, a power function relationship between the subgrade resilient modulus (*E*_0_) and *CBR* was obtained (i.e., the Shanping loess subgrade *E*_0_ = 0.70*CBR*^0.96^, correlation coefficient *R*^2^ = 0.726; Guangyuan loess subgrade *E*_0_ = 6.46*CBR*^0.48^, correlation coefficient *R*^2^ = 0.674; Heyun loess subgrade* E*_0_ = 0.07*CBR*^1.66^, and correlation coefficient *R*^2^ = 0.708). Fang et al.^26^ used unmodified fine-grained sulfate soil (clay and silt) in the Qinghai region as the raw material to perform laboratory tests on the resilient modulus and *CBR*. By analyzing and fitting a large amount of test data, it was found that there was a good fitting relationship between the resilient modulus (*E*_0_) and *CBR*_2.5_ under different test conditions (i.e., *E*_0_ = *aCBR*^b^). The correlation coefficient between *E*_0_ and non-flooded *CBR*_2.5_ is above 0.9, which is better than that between *E*_0_ and flooded *CBR*_2.5_. Deng et al.^27^ conducted an indoor repeated loading triaxial test and *CBR* test on the road performance index (dynamic resilient modulus and *CBR*) of coarse-grained chlorine saline soil, focusing on the influence of water and salt on the two design indices, and evaluated the feasibility of this type of saline soil as a subgrade filler. However, this study did not analyze the correlation between the two design indicators in detail.

These results have shown that the correlation studies of the subgrade resilient modulus and *CBR* mainly focus on the static resilient modulus (*E*_0_) and *CBR* of fine soil (such as loess, clay, and silt), and the relationship between them. However, there is little research on the dynamic resilient modulus of widely distributed four-phase saline soil, which has led to the abandonment of large areas of natural coarse-grained saline soil, especially coarse-grained chlorine saline soil, with good strength and stability. Considering the differences in particle composition, history, origin, scale, stable state, and degree of damage to highways in different regions, there are certain differences in engineering mechanical properties, resulting in different relationship models between the dynamic resilient modulus (*M*_R_) and *CBR* of the subgrade soil in different regions. Therefore, it is necessary to study the correlation between the dynamic resilient modulus and *CBR* of coarse saline soil to provide a simple and practical method for obtaining subgrade design parameters in saline soil areas.

Considering this, this study uses coarse-grained chlorine saline soil as the research object (coarse-grained saline soil in the following expression refers to chloride saline soil) and conducts experimental studies on the dynamic resilient modulus (*M*_R_) and *CBR* values of coarse-grained saline soil under different stress levels, water contents, and salt contents through repeated loading triaxial tests and *CBR* tests. The change law and correlation between the dynamic resilient modulus and load-bearing ratio under different test conditions were analyzed. Through statistical analysis of the test data, a prediction model for the dynamic resilient modulus concerning CBR was developed to accurately estimate the dynamic resilient modulus of coarse-grained chlorine saline soil. This model offers a simple and re-liable method for obtaining design parameters of saline soil subgrade in a reasonable manner. It holds significant reference value and importance in guiding the design and construction of saline soil subgrade.

## Materials and methods

### Test materials

Owing to regional differences in the original state, historical origin, abundance scale, and environmental conditions of the saline soil samples on site, it was impossible to accurately control its physical property indices, such as salt content, water content, and particle composition, by artificial means. The test material used in this study was coarse-grained chlorine saline soil artificially configured in the laboratory to reduce the influencing factors of the test and more accurately and comprehensively study the correlation between the dynamic resilient modulus of coarse-grained saline soil and its *CBR*.

The original soil sample was determined to be plain soil (salt content less than 0.1%) by soluble salt content. Its particle size composition and basic physical property indices are shown in Tables 1 and 2. According to the relevant norms^13^, the saline soil was classified according to the degree of salinization, and the salt content limit of NaCl was 0% (coarse-grained soil), 2% (weakly saline), 5% (medium-saline), and 8% (strongly saline). Coarse-grained chloride saline soil with the prescribed salt content was obtained by blending NaCl with well-graded coarse-grained soil using the mass method.

**Table 1:** Particle size composition of soil sample.

Filter aperture (mm)	Mass left on aperture (g)	Cumulative mass greater than aperture (g)	Mass percentage left on aperture (%)	Cumulative mass percentage greater than aperture (%)	Mass percentage less than aperture (%)
20	0.0	0.0	0.0	0.0	100
10	2521.4	2521.4	39.21	39.21	60.79
5	1130.1	3651.5	17.57	56.78	43.22
2	1130.2	4781.7	17.57	74.35	25.65
1	386.8	5168.5	6.01	80.36	19.64
0.5	671.9	5840.4	10.45	90.81	9.19
0.25	323.0	6163.4	5.02	95.83	4.17
0.075	227.6	6391.0	3.54	99.37	0.63
< 0.075	40.3	6431.3	0.63	100.00	0.00

**Table 2:** Basic physical properties of soil sample.

Soil source	Particle size *d* < 2 mm (%)	*d* < 20 mm (%)	$\rho_{{{\text{dmax}}}}$ (g cm^−3^)	*ω*_opt_ (%)	*C*_u_	*C*_c_	Gradation state	Soil classification
Nanyang, China	25.65	100	2.33	5.1	18.11	1.42	Well-graded	Gravelly soil

### Sample preparation

The sample was controlled with a unified compaction degree of 96% (the ratio of field density to indoor density) and prepared under different salt and water contents to study the evolution law of the dynamic resilient modulus and *CBR* value of coarse-grained chloride saline soil affected by water content and salt content. Samples with the same salt content were prepared with moisture contents of 4%, 5.1%, and 6%, with three parallel samples in each group. The test conditions are listed in Table 3.

**Table 3:** Test conditions.

Test number	1	2	3	4	5	6	7	8	9	10	11	12
Compactness (%)	96			96			96			96		
Salt content (%)	0			2			5			8		
Water content (%)	4	5.1	6	4	5.1	6	4	5.1	6	4	5.1	6

#### Triaxial test sample

**Figure 1:**
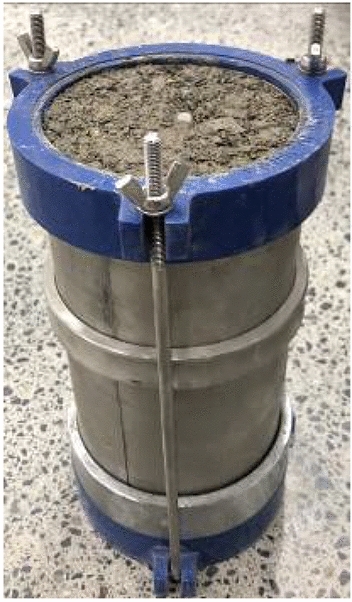
Triaxial specimen.

The sample size for the standard triaxial test has a height of 200 mm and a diameter of 100 mm. The samples were prepared in strict accordance with the relevant provisions for the dynamic resilient modulus test of subgrade soil in the Specifications for Design of Highway Subgrades (JTG D30-2015, China) and the Test Methods of Soils for Highway Engineering (JTG 3430-2020, China) (Fig. 1). To ensure a uniform degree of compaction for the entire sample, it was divided into eight layers in a split die. The soil sample quality was used to control the loose layup thickness of each layer to ensure that the compaction of each layer reached the target value. Hair drawing between the layers was simultaneously performed to ensure the integrity of the specimen.

#### CBR test sample

The sample for the *CBR* test was obtained using a heavy compaction test, and the sample size, compaction times, and number of layers were determined in accordance with the relevant provisions of the Test Methods of Soils for Highway Engineering (JTG 3430-2020, China). In this study, a compaction cylinder, with a height of 170 mm and a diameter of 150 mm, was used to prepare the samples. The test soil sample was prepared under optimal water content conditions, and the target compaction degree was controlled by the mass and volume. Comparison test groups with moisture content of *ω*_opt_ ± 1% were set to analyze the effect of humidity on the *CBR* of coarse-grained chlorine saline soil.

### Test methods

#### Dynamic resilient modulus test

**Figure 2:**
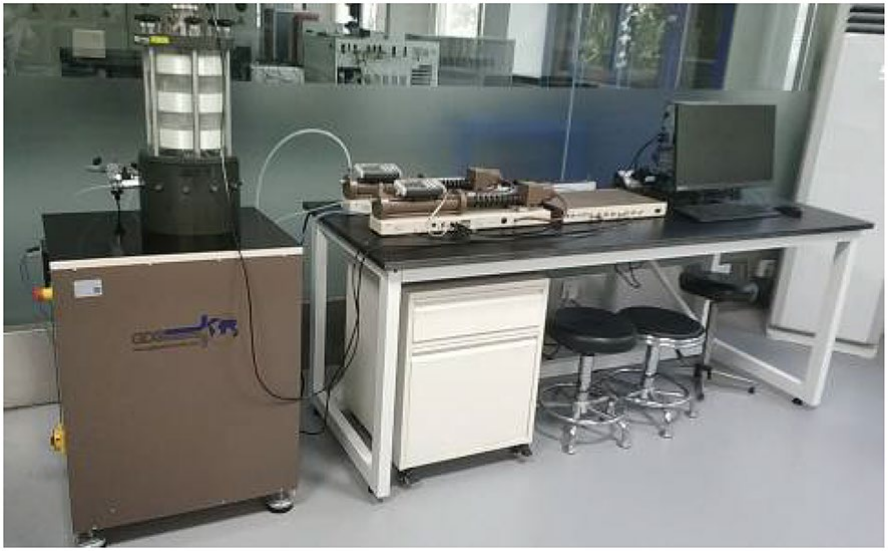
Dynamic triaxial test system.

A British GDSLAB dynamic triaxial test system (5 Hz/60 kN MinDyn) was used for the dynamic resilient modulus tests in this study. The triaxial pressure chamber has a height of 300 ± 2 mm and an inner diameter of 170 ± 2 mm (Fig. 2). This test adopted half-sine wave loading with a load frequency of 1 Hz, load holding time of 0.2 s, intermittent time of 0.8 s, and confining pressure of pneumatic loading.

Based on the structural characteristics of China's typical asphalt pavement and the stress state of the subgrade, Luo and Chen et al.^28,29^ proposed a dynamic triaxial stress loading sequence for subgrade coarse-grained soil with reference to the NCHRP 1–28^30^ test method (as shown in Table 4). The above stress-loading sequence was adopted to perform repeated loading triaxial tests in this study, and the operational procedures were strictly in accordance with the Chinese standard test methods stipulated in the Specifications for Design of Highway Subgrades and Test Methods of Soils for Highway Engineering. The dynamic resilient modulus of the coarse-grained chlorine saline soil was calculated according to Eq. (1).

1 $$M_{{\text{R}}} = \frac{{\sigma _{\text{d}} }}{{\varepsilon _{{\text{R}}} }},$$

where *M*_R_ is the dynamic resilient modulus (MPa), *σ*_d_ is the deviator stress (kPa), *σ*_d_ = *σ*_1_ − *σ*_3_, *σ*_1_ is the vertical stress, *σ*_3_ is the confining stress (generally *σ*_2_ = *σ*_3_), and *ε*_R_ is the mean axial rebound strain (0.001 mm).

**Table 4:** Triaxial loading sequence of coarse-grained soil (Unit: kPa).

Serial number	Confining stress (*σ*_3_)	Contact stress (0.2*σ*_3_)	Deviator stress (*σ*_*d*_)	Axial stress (*σ*_*max*_)	Number of loads
0- preload	30	6	60	66	1000
1	15	3	8	11	100
2	30	6	15	21	100
3	45	9	23	32	100
4	60	12	30	42	100
5	80	16	40	56	100
6	15	3	15	18	100
7	30	6	30	36	100
8	45	9	45	54	100
9	60	12	60	72	100
10	80	16	80	96	100
11	15	3	30	33	100
12	30	6	60	66	100
13	45	9	90	99	100
14	60	12	120	132	100
15	80	16	160	176	100

#### California bearing ratio (CBR) test

**Figure 3:**
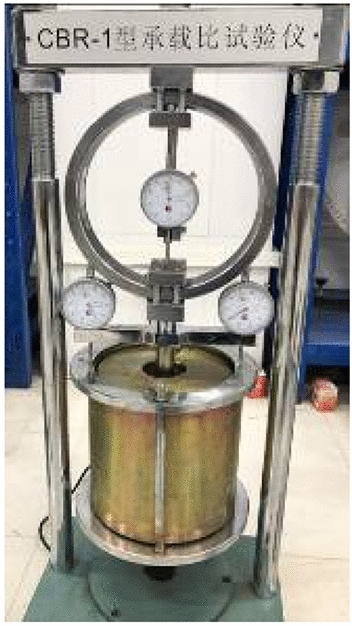
CBR-1 bearing ratio tester.

The *CBR* test in this study adopted the CBR-1 bearing ratio tester produced by the Chinese Nanjing Ningxi Soil Instrument Co., Ltd. (Fig. 3). Its penetration rod has a standard size H × D = 100 × 50 mm, the maximum load was 30 kN, and the lifting speed was 1 mm/min. In addition, it was necessary to use three dial indicators and their supporting shelves, four load plates (1.25 kg each), perforated plates, and other supporting-measuring equipment.

**Figure 4:**
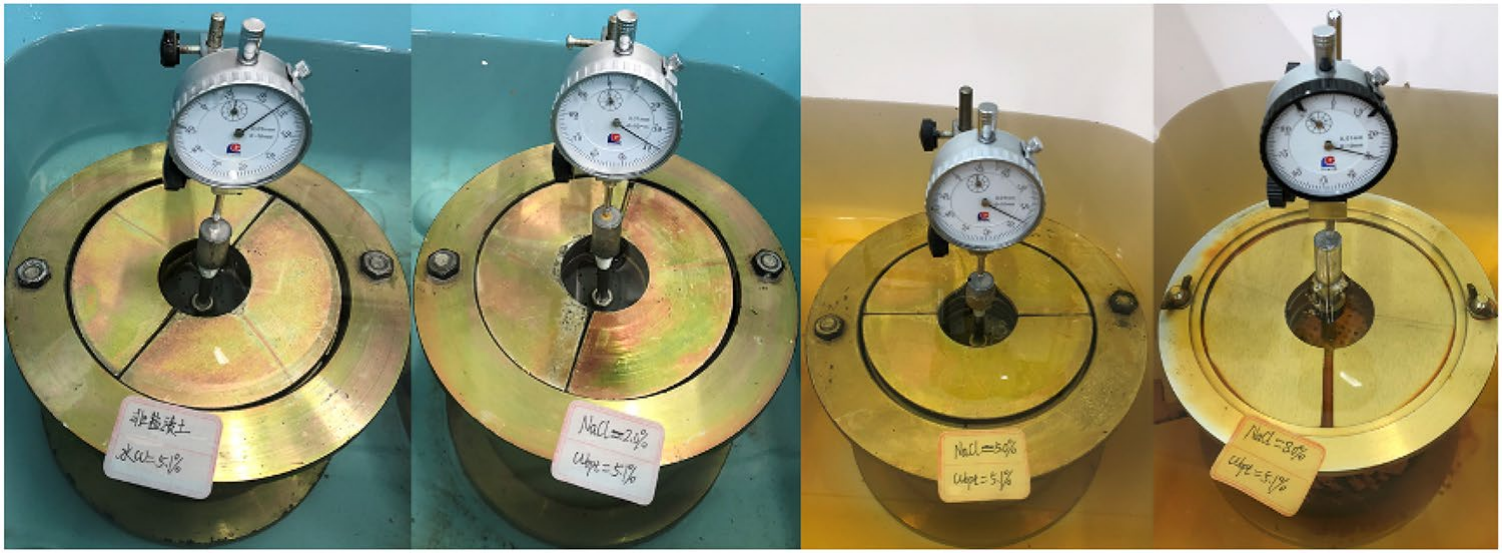
CBR sample in saturated water.

Considering the decrease in subgrade strength caused by rain and groundwater immersion, the *CBR* after 96 h of immersion is often used as the most unfavorable standard for subgrade filling in subgrade design. For the specific operating procedures of the sample immersion test (Fig. 4) and penetration test (Fig. 3), please refer to the T0134-2019 Bearing ratio (*CBR*) test in the Test Methods of Soils for Highway Engineering (JTG 3430-2020, China).

In this study, the dial meter readings were recorded at penetration depths of 30, 50, 100, 150, 200, 250, 300, 350, 400, 450, 500, 550, and 600 m (units = 0.01 mm). The stress-penetration curves of the samples under different test conditions were plotted using the penetration quantity and unit compressive stress as the transverse and longitudinal coordinates, respectively (Fig. 7). Based on the calculation and value regulation of the *CBR* issued by the latest Test Methods of Soils for Highway Engineering (JTG 3430-2020) of China, the ratios of unit pressure and standard pressure corresponding to 2.5 and 5.0 mm penetration were calculated, respectively (as percentages). The larger value of the two was considered as the *CBR* of the coarse-grained chlorine saline soil, and the specific calculation method is shown in Eqs. (2) and (3).

2 $$CBR_{{2.5}} = \frac{{P_{{2.5}} }}{{7000}} \times 100,$$

3 $$CBR_{{5.0}} = \frac{{P_{{5.0}} }}{{10500}} \times 100,$$

where *CBR*_2.5_ and *CBR*_5.0_ are the bearing ratio (%) when the penetration depth is 2.5 and 5.0 mm, respectively, and *P*_2.5_ and *P*_5.0_ are the corresponding unit pressure values (kPa) when the penetration volume is 2.5 and 5.0 mm, respectively.

In addition to the determination of *CBR* for coarse-grained chloride saline soil with optimal moisture content, this study also tested the *CBR* for *ω*_opt_ ± 1% moisture content to compare the influence of humidity on *CBR* value of coarse-grained chloride saline soil.

## Results and discussion

**Figure 5:**
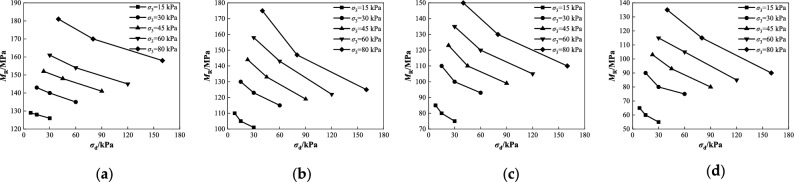
Relationship between *M*_R_ and *σ*_d_ under different confining pressures at 5.1% water content: (**a**) *Z* = 0%; (**b**) *Z* = 2%; (**c**) *Z* = 5%; (**d**) *Z* = 8%.

**Figure 6:**
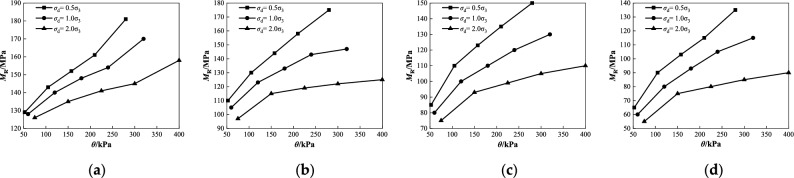
Relationship between *M*_R_ and *θ* under different partial stresses at 5.1% water content: (**a**) *Z* = 0%; (**b**) *Z* = 2%; (**c**) *Z* = 5%; (**d**) *Z* = 8%.

**Figure 7:**
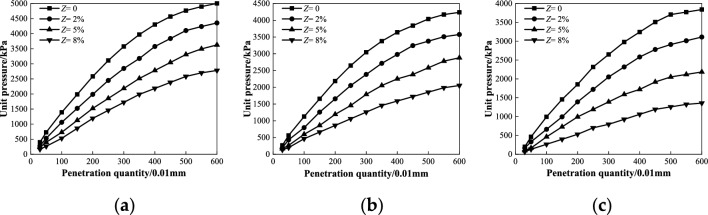
Relationship between unit pressure and penetration quantity under different salt contents at degree of compaction *k* = 96%: (**a**) *ω* = 4%; (**b**) *ω* = 5.1%; (**c**) *ω* = 6%.

**Figure 8:**
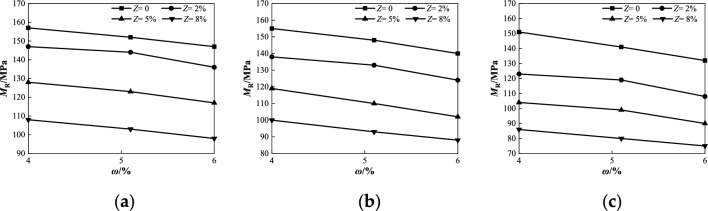
Relationship between *M*_R_ and *ω* under different salt contents at confining pressure 45 kPa: (**a**) *σ*_d_ = 23 kPa; (**b**) *σ*_d_ = 45 kPa; (**c**) *σ*_d_ = 90 kPa.

**Figure 9:**
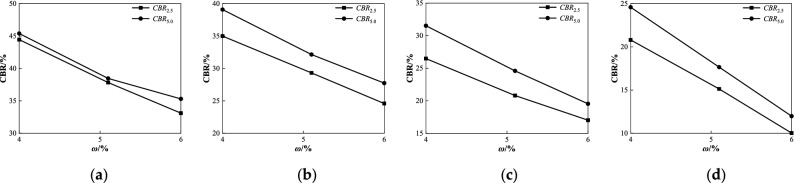
Relationship between *CBR* and *ω* under different penetrations at degree of compaction *k* = 96%: (**a**) *Z* = 0%; (**b**) *Z* = 2%; (**c**) *Z* = 5%; (**d**) *Z* = 8%.

**Figure 10:**
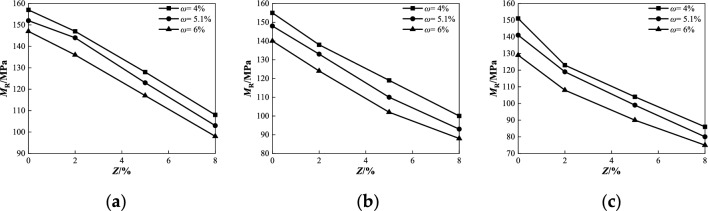
Relationship between *M*_R_ and *Z* under different water contents at confining pressure 45 kPa: (**a**) *σ*_d_ = 0.5*σ*_3_ = 23 kPa; (**b**) *σ*_d_ = 1.0*σ*_3_ = 45 kPa; (**c**) *σ*_d_ = 2.0*σ*_3_ = 90 kPa.

**Figure 11:**
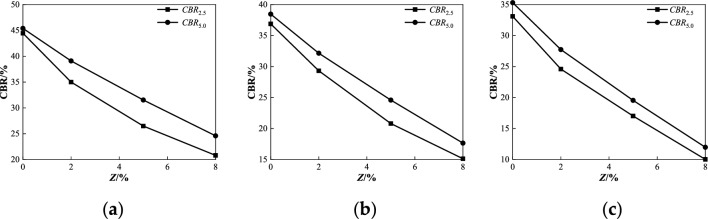
Relationship between *CBR* and *Z* under different penetrations at degree of compaction *k* = 96%: (**a**) *ω* = 4%; (**b**) *ω* = 5.1%; (**c**) *ω* = 6%.

To analyze the evolution law of the dynamic resilient modulus and *CBR* value of coarse-grained chloride saline soil affected by stress conditions, water content, and salt content, the compaction degree of the selected test samples was 96%, and the test results are shown in Figs. 5, 6, 7, 8, 9, 10 and 11. To facilitate expression and interpretation, the water content, salt content, confining stress, deviator stress, bulk stress, and dynamic resilient modulus were expressed by *ω*, *Z*,* σ*_3_, *σ*_d_, *θ*, and *M*_R_ in this study, respectively. In addition, there was a multiple relationship between the deviator stress and the confining stress (*σ*_d_ = 0.5*σ*_3_, 1.0*σ*_3_, 2.0*σ*_3_) and *θ* = *σ*_1_ + *σ*_2_ + *σ*_3_.

### Influence of stress conditions on dynamic resilient modulus and penetration quantity

As shown in Fig. 5, when the optimal moisture content was 5.1%, under different salt conditions, the dynamic resilient modulus of the coarse saline soil with different confining pressures decreased with an increase in the deviator stress. With an increase in deviator stress, when *Z* = 0, the decreases in the dynamic resilient modulus *M*_R_ under various confining pressures were 2.3%, 5.6%, 7.2%, 9.9%, and 12.7% (Fig. 5a). When *Z* = 2%, the decrease in *M*_R_ increased to 8.2%, 11.5%, 17.4%, 22.8%, and 28.6% (Fig. 5b). When *Z* = 5%, the reduction in *M*_R_ ranged from 11.7 to 26.7% (Fig. 5c). When *Z* = 8%, the reduction of *M*_R_ Increases to 15.4–33.3% (Fig. 5d). The greater the deviator stress, the greater the reduction in the dynamic resilient modulus, and the higher the salt content in the coarse saline soil, the more obvious the influence of the deviator stress on the dynamic resilient modulus. The microscopic mechanism of this phenomenon can be explained as follows. When the salt content *Z* in the soil is less than the limit salt content (i.e., corresponding to the saturated state of the salt solution, the limit salt content is approximately 10%)^31,32^, the ion concentration after dissolution increases with increasing salt content, the thickness of the double electric layer increases, the lubrication effect of sodium ions gradually strengthens, and the shear strength of the chloride saline soil gradually decreases. The shear effect caused by the increase in the deviator stress was greater than the lateral effect caused by the shear strength and confining pressure of the soil in the late loading period, leading to dislocation movement and rearrangement of the soil particles, and the dynamic resilient modulus gradually decreased.

As shown in Fig. 6, when the humidity is the optimal moisture content of 5.1%, under different salt content conditions, the dynamic resilient modulus of coarse-grained saline soil with different deviator stresses increases as the bulk stress increases. As the bulk stress increases, when *Z* = 0, as shown in Fig. 6a, the dynamic resilient modulus *M*_R_ under different deviator stresses increases by 40.3%, 32.8%, and 25.4%. As shown in Fig. 6b, when *Z* = 2%, *M*_R_ increased by 59.1%, 40.0%, and 28.9%, respectively. As shown in Fig. 6c, when *Z* = 5%, *M*_R_ increased by 76.5%, 62.5%, and 46.7%, respectively. As shown in Fig. 6d, when *Z* = 8%, *M*_R_ increased by 107.7%, 91.7%, and 63.6%, respectively. The experimental observation shows that the smaller the deviator stress, the larger the increase in the dynamic resilient modulus caused by the increase in bulk stress. The higher the salt content of the coarse saline soil, the more evident the influence of bulk stress on its dynamic resilient modulus. Based on the analysis, when the deviator stress is constant, the increase in bulk stress is due to the strengthening of the confining pressure *σ*_3_ on the transverse deformation of the sample, the increase in soil stiffness, and the gradual increase in the dynamic resilient modulus.

As shown in Fig. 7, the amount of penetration with different salt contents increased with an increase in the unit compressive stress when the degree of compaction was 96% under various humidity conditions. Moreover, the penetration quantity of the low-salt content (*Z* ≤ 2%) increases nonlinearly, whereas that of the high-salt content (*Z* > 2%) increases approximately linearly. When the unit compressive stress was constant, the penetration quantity of the sample with the same humidity increased with increasing salt content. When *Z* increases from 2 to 8%, the vertical local compressive stress of the coarse saline soil with different humidities gradually decreases, and the decreasing range increases from 13.89%, 16.39%, and 21.43% to 45.83%, 54.10%, and 66.07%, respectively. This indicates that the penetration quantity with a smaller salt content is more weakly affected by unit pressure, and the influence of unit compressive stress on the penetration quantity becomes gradually obvious with an increase in salt content, especially for samples with high salt content. This shows that the unit compressive stress has a significant influence on the penetration of a sample with a high salt concentration. In other words, the higher the salt content, the smaller is the resistance of the coarse saline soil to local loading deformation. The test law can be analyzed as follows: after the coarse saline soil is immersed in water, the salt in the soil is easily dissolved in water and loses water, and the soil void increases further. Consequently, the compactness of the sample decreased, the compressibility increased, and the deformation resistance decreased, which is consistent with the above law that the dynamic resilient modulus is affected by the vertical deviator stress.

### Influence of water content on dynamic resilient modulus and CBR value

As shown in Fig. 8, when the confining pressure was 45 kPa, the dynamic resilient modulus of coarse saline soil with different salt contents gradually decreased with increasing water content under various deviator stress conditions. When the water content increases from 4 to 6% and *σ*_d_ = 23 kPa, the dynamic resilient modulus *M*_R_ with different salt content decreases by 6.4%, 7.5%, 8.6%, and 9.3%, respectively (Fig. 8a). When *σ*_d_ = 45 kPa, *M*_R_ decreases by 9.7%, 10.1%, 13.3%, and 12.0%, respectively (Fig. 8b). When *σ*_d_ = 90 kPa, *M*_R_ decreases by 12.6%, 12.2%, 13.5%, and 12.8%, respectively (Fig. 8c). The analysis of the test data shows that when the salt content is constant, the decrease of the dynamic resilient modulus increases with the increase of the deviator stress, and the maximum difference of the reduction is in the range of 3.5–6.2%. This is because an increase in water causes the water film between the soil particles to thicken and the salt dissolved in the water destroys the salt skeleton in the soil. In particular, under the condition of high salt content, with an increase in water content, the lubrication effect of the salt solution was enhanced, and the friction strength between coarse particles of soil was weakened, resulting in a gradual decrease in the dynamic resilient modulus.

As shown in Fig. 9, when the degree of compaction is 96%, the *CBR* value of coarse-grained chlorine saline soil with different penetration quantities decreases with an increase in water content under different salt concentration conditions, and the *CBR* value of the 5 mm penetration quantity is greater than that of the 2.5 mm (i.e., the *CBR* value corresponding to a 5 mm penetration quantity is the bearing ratio of the soil sample tested in this study). With an increase in the water content in the molded sample, the CBR value under each salt concentration condition decreased by 10.09%, 11.15%, 11.98%, and 12.61%. When the salt content increases from 2 to 8% (as shown in Fig. 9b,c, and d), the difference between the *CBR* value of the 2.5 mm penetration quantity and the *CBR* value of 5 mm tends to shrink with the increase in water content, and both gradually decrease. This test rule shows that the *CBR* value of coarse-grained chlorine saline soil is significantly affected by the humidity before sample molding. The higher the moisture content in the coarse chlorine saline soil, the smaller the *CBR* value measured after immersion. The reasons for this test phenomenon are derived from the water film theory^33^. The thickening of the water-film diffusion layer weakened the suction and effective connection between the soil particles, and the shear strength of the soil decreased, resulting in the failure of the sample to achieve the expected compactness after compaction. However, this is mainly because, after the sample is immersed in water, the particle framework of the soil is damaged again by water and salt migration, and the plasticity is further enhanced. Therefore, the strength attenuation value of the coarse-grained saline soil with a greater moisture content is more significant after soaking in water.

### Influence of salt content on dynamic resilient modulus and CBR value

As shown in Fig. 10, when the confining pressure was 45 kPa, the dynamic resilient modulus *M*_R_ of coarse saline soil with different water contents gradually decreased as the salt content increased under various deviator stress conditions. When the salt content *Z* increases from 0 to 8%, and *σ*_d_ = 23 kPa (Fig. 10a), the dynamic resilient modulus *M*_R_ of different water content decreases by 31.2%, 32.2%, and 33.3%, respectively. As shown in Fig. 10b, when *σ*_d_ = 45 kPa, *M*_R_ decreases by 35.5%, 37.2%, and 37.1%, respectively. As shown in Fig. 10c, when *σ*_d_ = 90 kPa, *M*_R_ decreases by 43.0%, 43.3%, and 41.9%, respectively. The results indicate that as the salt content increases, the decline in dynamic resilient modulus of coarse-grained saline soils with varying water content tends to rise, and the maximum difference is in the range of 1.4–2.1%. When the deviator stress increases from 23 to 90 kPa, the decrease in the dynamic resilient modulus gradually increased with the increase in salt content, and the maximum difference in the decrease range was 8.6–11.8% under different humidity conditions. The primary reason is that the rise in sodium chloride causes expansion of the salt skeleton within the soil. Upon contact with water, more salt skeletons dissolve, thereby increasing the space between soil particles and consequently enhancing soil compressibility. Simultaneously, the increase in salt solution concentration promotes lubrication among soil particles, making it easier for soil particles to slip or rearrange under cyclic loading, resulting in plastic deformation, which results in the decline of the soil resilient deformation ability, consistent with existing research conclusions^34–36^.

As shown in Fig. 11, when the degree of compaction was 96%, the *CBR* value of the coarse saline soil samples formed under various humidity conditions decreased with an increase in salt content after water immersion. With the increasing salt content of the samples, the *CBR* values under different humidity conditions decreased from 45.40%, 38.46%, and 35.31% to 24.59%, 17.66%, and 11.98%, respectively. In addition, with the increase of salt content, the difference between *CBR* value of 5.0 mm penetration and *CBR* value of 2.5 mm penetration increased first and then decreased, and the maximum difference between the two was 5.04%, 3.78%, and 3.15%, respectively. With an increase in salt content, the decrease in the *CBR* value of coarse saline soil with different moisture contents tends to increase, with a maximum decrease of 23.33% (corresponding to the condition of 6% moisture content). This test phenomenon verified that the dynamic resilient modulus was affected by the chloride content. That is, with the increase of salt content (*Z* ≥ 2%)^36,37^, when the soil is immersed in water for a short time and there is not much water, part or all of the chlorine salts in the soil are dissolved in water, and the salt skeleton is destroyed, which increases the space between the soil particles and gradually increases the dissolution deformation rate under the soil body weight and load. However, when the coarse saline soil is immersed for a long time (4 days and nights) and the amount of water immersed is large, resulting in seepage, the chlorine salt is fully dissolved with the water loss but also removes part of the soil particles in the soil, resulting in latent erosion and a further increase in the void in the soil. As a result, additional deformation (i.e., latent erosion deformation, which is the main component of solution subsidence deformation of coarse-grained saline soil) is generated in the soil, resulting in a gradual decrease in the strength of the chlorinated saline soil, which is more consistent with the existing research results of chloride salt solution subsidence^38–40^.

In summary, the dynamic resilient modulus (*M*_R_) and *CBR* values of the coarse-grained chloride saline soil were similar under the influences of stress, water, and salt, and there was a good mechanical correlation between them. The measured values under different test conditions were sorted and statistically analyzed, as shown in Table 5, to further clarify the specific relationship between the dynamic resilient modulus and *CBR*.

**Table 5:** Dynamic resilient modulus and *CBR* test values at the compaction degree of 96%.

Test conditions	Dynamic resilient modulus* M*_R_ (MPa)				*CBR* after 96 h saturation (%)		
*Z* (%)	*ω* (%)	Min value	Max value	Average value	*CBR*_2.5_	*CBR*_5.0_	*CBR*
0	4	131.12	185.85	153.73	44.45	45.40	45.40
	5.1	125.68	181.17	147.41	36.89	38.46	38.46
	6	118.25	174.38	138.47	33.10	35.31	35.31
2	4	100.43	179.86	133.35	35.00	39.09	39.09
	5.1	101.37	175.29	130.24	29.32	32.16	32.16
	6	95.38	159.76	121.13	24.59	27.74	27.74
5	4	80.42	160.36	114.67	26.48	31.53	31.53
	5.1	75.19	149.92	108.33	20.81	24.59	24.59
	6	69.81	140.42	100.61	17.02	19.55	19.55
8	4	60.33	140.44	95.83	20.81	24.59	24.59
	5.1	54.87	134.75	89.73	15.13	17.66	17.66
	6	51.05	130.39	85.42	10.03	11.98	11.98

Annotation: (1) The minimum value is the test value of the dynamic resilient modulus of the sample under the most unfavorable stress conditions (i.e., *σ*_3_ = 15 kPa, *σ*_d_ = 30 kPa). (2) The maximum value is the test value of the dynamic resilient modulus of the sample under optimal stress conditions (i.e., *σ*_3_ = 80 kPa, *σ*_d_ = 40 kPa). (3) The average value is the average value of the test value of the dynamic resilient modulus of the sample under the 15 stress conditions in Table 4.

According to the test results in Table 5, the average dynamic resilient modulus under each test condition ranges from 85.42 to 153.73 MPa, with the minimum value recorded at 51.05 MPa. These values comply with the minimum resilient modulus requirements for saline soil subgrade specified in the Guidelines for Highway Design and Construction in the Saline Soil Area of China^41^. Thus, it is necessary to build high-grade and first-class highways in areas with strongly saline soil and over saline land, and the subgrade resilient modulus cannot be less than 35 MPa. It also meets the requirements of the minimum resilient modulus of the top surface of the road bed in the light traffic load class stipulated in the current Chinese road design codes (JTG D50 and D40). The test results showed that the saturated-water *CBR* value under different working conditions was greater than 11.98%, exceeding the minimum value of 8% required by the current subgrade design code and subgrade Construction Technical Code. The roadworthiness requirements of the current subgrade design and construction codes are consistent with respect to the strength of the fillers and analysis based on the deformation resistance of the subgrade. Therefore, a certain mechanical correlation exists between the dynamic resilient modulus of coarse chloride saline soil and its *CBR* value.

## Prediction model of dynamic resilient modulus based on *CBR* value

### Determination of the prediction model of dynamic resilient modulus

According to the analysis of the above test results, the evolution law of the dynamic resilient modulus and *CBR* value of the coarse-grained chloride saline soil under the influence of stress, water, and salt was the same, which is consistent with the theoretical conclusion that the tangent modulus of the penetration curve was approximately the elastic modulus in the *CBR* test^1^. Therefore, the form of the model formula should first be determined when determining a prediction model for the dynamic resilient modulus to accurately reflect the internal relationship between them.

The relationship between the soil resilient modulus and *CBR* value has always been a concern for highway builders worldwide. Initially, researchers in the Netherlands, the United States, France, the United Kingdom, and other European and American countries conducted a large number of tests and studies on the relationship between the soil resilient modulus and the *CBR* value and obtained an approximate relationship between the subgrade soil *CBR* and the resilient modulus in different countries^25^. The specific model formulae are listed in Table 6.

**Table 6:** Relationship between foreign soil resilient modulus and *CBR* value.

Encoding	Data source	Model formula	Model characteristics and test types
1	Dutch oil company *Shell*	*E*_0_ = 10*CBR*	*E*_0_ -Dynamic elastic modulus; Field test
2	Dutch oil company *Shell*	*E*_0_ = 5*CBR*	*E*_0_ -Static resilient modulus; Field test
3	American Hcukclom	*E*_0_ = 6.5*CBR*	*E*_0_ -Static resilient modulus; Field and Indoor
4	American Asphalt Institute	*E*_0_ = 10.5*CBR*	*E*_0_ -Static resilient modulus; Field and Indoor
5	Kentucky, USA	*E*_0_ = 12.4*CBR*^0.688^	*E*_0_ -Static resilient modulus; Field test
6	France	*E*_0_ = 2–5*CBR*^0.555^	*E*_0_ -Static resilient modulus; Field and Indoor
7	French Handbook for Road Design	*E*_0_ = 5*CBR*^0.555^	*E*_0_ -Static resilient modulus; Field and Indoor
8	Jcuffray Bacheler of France	*E*_0_ = 6.5*CBR*^0.65^	*E*_0_ -Static resilient modulus; Indoor test
9	British *TRRL*	*E*_0_ = 17.6*CBR*^0.54^	*E*_0_ -Static resilient modulus; Field test
10	Japan Road Corporation	*E*_0_ = 2–4*CBR*^0.555^	*E*_0_ -Static resilient modulus; Field test
11	Morocco	*E*_0_ = 8.9*CBR*^0.85^	*E*_0_ -Static resilient modulus; Field and Indoor
12	Czech Republic	*E*_0_ = 12.1*CBR*^0.70^	*E*_0_ -Static resilient modulus; Field and Indoor

Many Chinese researchers have conducted numerous tests and studies on the relationship between the resilient modulus and *CBR* of eight representative subgrade soils in Xinjiang, expansive soil and clay in Guangxi, loess in Shanxi and Shaanxi, red clay in Guizhou, clay in Inner Mongolia, and clay in Heilongjiang. A method for calculating the resilient modulus based on the CBR value was proposed, and prediction models^25,42^ were obtained. The model forms are presented in Table 7.

**Table 7:** Relationship between Chinese soil resilient modulus and *CBR* value.

Encoding	Data source	Model formula	Model characteristics and test types
1	Xinjiang silt, gravel soil, desert sand	*E*_0_ = 3–5*CBR*	*E*_0_ -Static resilient modulus; Field and Indoor
2	Guangxi expansive soil, clay	*E*_0_ = 2.2–17.62*CBR*^0.46–1.08^	*E*_0_ -Static resilient modulus; Field and Indoor
3	Shanxi loess	*E*_0_ = 0.07–6.46*CBR*^0.48–1.66^	*E*_0_ -Static resilient modulus; Field and Indoor
4	Shaanxi loess	*E*_0_ = 1.60–12.0*CBR*^0.42–1.12^	*E*_0_ -Static resilient modulus; Field and Indoor
5	Guizhou red clay	*E*_0_ = 6.70–7.87*CBR*^0.78–0.82^	*E*_0_ -Static resilient modulus; Field test
6	Shanghai clay soil	*E*_0_ = 7.90–15.86 *CBR*^0.59–0.91^	*E*_0_ -Static resilient modulus; Indoor test
7	Inner Mongolia clay	*E*_0_ = 6.70–7.03*CBR*^0.78–0.87^	*E*_0_ -Static resilient modulus; Field and Indoor
8	Heilongjiang clay soil	*E*_0_ = 2.6–4.9*CBR*^0.38–1.09^	*E*_0_ -Static resilient modulus; Indoor test
9	Guangdong high liquid limit clay	*E*_0_ = 2.1–4.0*CBR*^0.39–1.01^	*E*_0_ -Static resilient modulus; Indoor test
10	Beijing silty clay	*E*_0_ = 2.2–6.4*CBR*^0.29–1.07^	*E*_0_ -Static resilient modulus; Indoor test
11	Highway Research Institute	*E*_0_ = 2.44–14.4*CBR*	*E*_0_ -Static resilient modulus; Field and Indoor

The relevant domestic and foreign investigations in Tables 6 and 7 show that the relationship between the soil resilient modulus and its *CBR* value is *E*_0_ = *k*_1_*CBR*^*k*^_2_. The current Specifications for Design of Highway Subgrades (JTG D30-2015) in China clearly provides a prediction model for estimating the resilient modulus of subgrade soil based on the *CBR* of the subgrade soil, as shown in Eqs. (4) and (5).

4 $$M_{R} = 17.6CBR^{{0.64}} \left( {2\% < CBR \le 12\% } \right)$$

5 $$M_{R} = 22.1CBR^{{0.55}} \left( {12\% < CBR < 80\% } \right)$$

This can provide an effective basis and scientific reference for the preliminary road design to be consistent with the mechanical state of the road subgrade. Based on previous studies’ results and the test data in this study, the prediction model of the dynamic resilient modulus of coarse-grained chloride saline soil established based on the *CBR* value is shown in Eq. (6).

6 $$M_{{\text{R}}} = {k_{1}}CBR^{{k_{2}}},$$

where *M*_R_ is the dynamic resilient modulus (MPa) and *CBR* is the *CBR* value (%) after 96 h of immersion. *k*_1_ and *k*_2_ are model parameters greater than zero.

### Regression analysis of model parameters

To accurately determine the relevant parameters of the prediction model, regression analysis was performed on the average dynamic resilient modulus of the coarse-grained chloride saline soil compacted to a degree of 96% (Table 5) (That is, the average value under the 15 stress conditions in Table 4), along with the CBR value after 96 h of water saturation. The resulting model parameters *k*_1_ and *k*_2_, as well as the error results, are presented in Table 8.

**Table 8:** Results for parameter regression analysis of dynamic resilient modulus prediction model.

Test conditions		*k*_1_ = 21.063, *k*_2_ = 0.516		*R*^2^ = 0.893	
*Z* (%)	*ω* (%)	Measured Average *M*_R_ (MPa)	Predicted Average *M*_R_ (MPa)	Residual error	Error (%)
0.0	4.0	153.73	150.86	2.87	1.87
	5.1	147.41	138.49	8.92	6.05
	6.0	138.47	132.51	5.96	4.31
2.0	4.0	133.35	139.65	6.30	4.73
	5.1	130.24	126.27	3.97	3.05
	6.0	121.13	117.00	4.13	3.41
5.0	4.0	114.67	124.98	10.31	8.99
	5.1	108.33	109.94	1.61	1.49
	6.0	100.61	97.66	2.95	2.93
8.0	4.0	95.83	109.94	14.11	14.73
	5.1	89.73	92.66	2.93	3.27
	6.0	85.42	75.86	9.56	11.19

**Figure 12:**
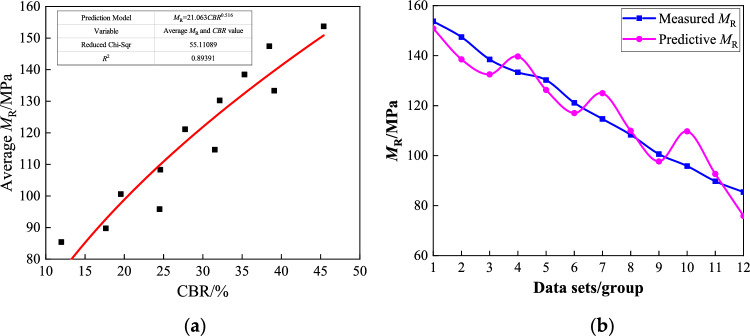
Relationship between average dynamic resilient modulus and *CBR* value: (**a**) Average* M*_R_ and *CBR*; (**b**) Measured *M*_R_ and predicted *M*_R_.

The results of the regression analysis in Table 8 show that the model parameters *k*_1_ = 21.063 and *k*_2_ = 0.516 are consistent with the formula parameters recommended in the current subgrade design code in China (Eq. (5)), and most of the CBR values measured in the test fall within the range of 12–80%. There was a high correlation coefficient (*R*^2^ = 0.893) between the established prediction model and the test results. The predicted value was in good agreement with the measured value, with an overall error within 10% and a maximum error of no more than 15%, as shown in Fig. 12.

### Model accuracy verification

**Figure 13:**
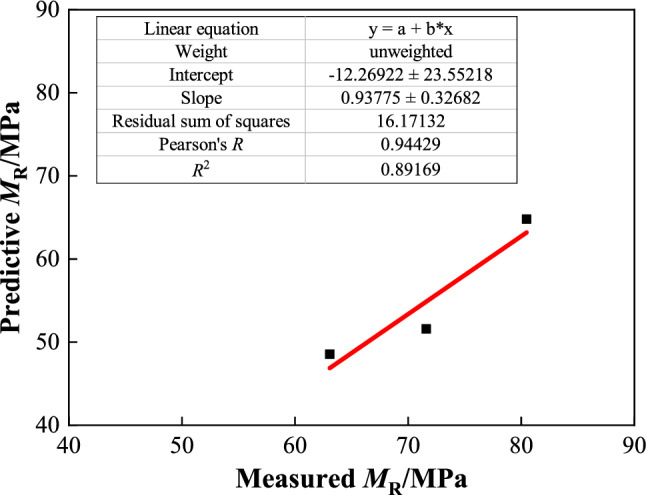
Correlation between predicted and measured values based on CBR value.

To verify the accuracy of the prediction model, another soil sample with a salt content *Z* = 10% (oversaline soil) was prepared, and the compaction degree was kept constant. Dynamic triaxial and *CBR* tests were conducted under three humidity conditions with water contents of 4.0%, 5.1%, and 6.0%. Average dynamic resilient modulus values measured were 80.47, 71.60, and 63.07 MPa, and the average *CBR* values measured were 8.83%, 5.67%, and 5.04%. The measured results were compared with the predicted values of the model (Fig. 13).

As shown in Fig. 13, the measured dynamic resilient modulus of the coarse-grained chloride saline soil with a salt content of 10% had a good correlation with the predicted value based on the *CBR*. The accuracy of the prediction model based on the *CBR* value formed using Eq. (6) was above 0.891, and the error between the measured and predicted values was small. The prediction model of the dynamic resilient modulus based on the *CBR* value is highly reliable and can effectively predict the dynamic resilient modulus of chloride saline soil under other environmental conditions in the prediction area.

## Conclusions

Repeated loading triaxial tests and *CBR* tests were carried out to study the correlation between the dynamic resilient modulus (*M*_R_) and *CBR* values of coarse-grained chloride saline soil, and the development law of the two mechanical indices under the influence of stress, water content, and salt content were discussed. Based on the correlation between the two revealed by experimental observations, a simple and reliable prediction model for the dynamic resilient modulus was proposed. The main conclusions of this study are as follows:

1. The *M*_R_ and *CBR* values of coarse-grained chloride saline soil were significantly dependent on stress. *M*_R_ gradually increased as the confining pressure and bulk stress increased, but significantly decreased with an increase in the deviating stress when the confining pressure was constant. The *CBR* value increased with an increase in the unit compressive stress, corresponding to a penetration quantity of 5.0 mm. An increase in the bulk stress caused an increase in the dynamic resilient modulus, which was caused by the lateral constraint of the confining pressure. Coarse-grained saline soil with high salt content has less resistance to local compressive stress after being saturated with water because chlorine salts are easily dissolved in water and are lost with water, resulting in insufficiently compacted soil samples and increased compressibility. The effect of stress on the two mechanical indices is the same, which is consistent with the conclusion that the tangent modulus of the penetration curve is approximately equal to the elastic modulus in the theoretical *CBR* test.
2. The dynamic resilient modulus (*M*_R_) and *CBR* values of the coarse-grained chloride saline soil were significantly affected by water and salt, and the influence of salt was more significant than that of water. Under the same stress, with an increase in water and salt content, the dynamic resilient modulus and *CBR* value after water saturation gradually decrease. With the increase of water content and salt content, the dynamic resilient modulus respectively decreases in the range of 6.4–13.5% and 31.2–43.3% (*σ*_3_ = 45 kPa), while the maximum decrease of *CBR* value is 12.61% and 23.33% (i.e., when the corresponding test conditions are *Z* = 8%, the water content is increased by 2%; salt content increases by 8% when *ω* = 6%). Analysis of the test data showed that the evolution laws of *M*_R_ and *CBR* were similar under the influence of water and salt, which further revealed a good mechanical correlation between them.
3. By investigating and analyzing the existing prediction models, it was determined that the typical model form between the dynamic resilient modulus and the *CBR* value is *E*_0_ = *k*_1_*CBR*^*k*^_2_. Based on this model, a regression analysis was performed on the test data, and the obtained model parameters (parameters *k*_1_ = 21.063 and *k*_2_ = 0.516) were consistent with the recommended formula parameters in the current subgrade design code in China. The error analysis showed that the predicted value was in good agreement with the measured value, and the error was generally less than 10%. The prediction model had high accuracy (*M*_R_ = 21.06*CBR*^0.52^ and correlation coefficient *R*^2^ = 0.893).

The study results accurately and comprehensively reflected the evolution law of the dynamic resilient modulus and *CBR* of coarse-grained chloride saline soil under the influence of load, moisture, and salt, and revealed a good correlation between them. The prediction model based on *CBR* is convenient for accurately predicting the dynamic resilient modulus of coarse chloride saline soils. The prediction model can provide a simple and reliable method for the reasonable selection of design parameters in the design process of roadbeds in saline soil areas. Because the prediction model of the dynamic resilient modulus established in this study was only obtained based on the test data of a graded coarse chloride saline soil, conducting testing research on the dynamic resilient modulus and *CBR* of coarse saline soil with multiple gradations in the future to verify and revising the prediction model in this study to improve its reliability and practical engineering application value are necessary.

## Data Availability

Data is available on request from the corresponding author.
